# Comparing the extraction methods, chemical composition, phenolic contents and antioxidant activity of edible oils from *Cannabis sativa* and *Silybum marianu* seeds

**DOI:** 10.1038/s41598-022-25030-7

**Published:** 2022-11-29

**Authors:** Monika Kalinowska, Anna Płońska, Magdalena Trusiak, Ewelina Gołębiewska, Anna Gorlewska-Pietluszenko

**Affiliations:** 1 Department of Chemistry, Biology and Biotechnology, Institute of Civil Engineering and Energetics, Faculty of Civil Engineering and Environmental Science, Bialystok University of Technology, Wiejska 45E Street, 15-351 Bialystok, Poland; 2 Department of Plant Physiology, Genetics and Biotechnology, University of Warmia and Mazury in Olsztyn, M Oczapowskiego 1A, 10-721 Olsztyn, Poland; 3 Olejowe Smaki, Felkowo 15, 18-106 Turośń Kościelna, Poland

**Keywords:** Plant sciences, Chemistry

## Abstract

In the study the cold-pressed, natural (unfiltered, unrefined) vegetable oils: hemp and milk thistle seed oils were tested for their chemical composition and antioxidant properties. The physico-chemical parameters, content of saturated and unsaturated fatty acids were determined. Solid phase extraction and simple extraction with the use of methanol, ethanol, 80% methanol, 80% ethanol were used to obtain the extracts for the analysis of antioxidant activity and phenolic compounds in oils. The composition of phenolic compounds was studied by means of high-performance liquid chromatography (HPLC–DAD) and spectrophotometric test with the Folin-Ciocalteu reagent. The antioxidant property of extracts was established by means of the following methods: with the DPPH^•^ (2,2-diphenyl-1-picrylhydrazyl) radical, ABTS^•+^ (2,2-azino-bis(3-ethylbenzothiazoline-6-sulfonic acid) cation radical, FRAP (ferric ion reducing antioxidant parameter) and CUPRAC (cupric-reducing antioxidant capacity). Moreover the influence of chlorogenic acid on the inhibition of lipid peroxidation process in the hemp and milk thistle seed oils was also investigated. The tested oils showed different antioxidant properties which was related to the their different chemical composition. The main phenolic compounds present in hemp seed oil were vanillic, ferulic and *p*-coumaric acids, (-)epicatechin, catechin, kaempferol and procyanidin B2, whereas in milk thistle seed oil—catechins, procyanidin B2, procyanidin C1, *p*-coumaric acid, phloridzin, quercetin, protocatechuic acid, kaempferol, and syringic acid. The methanolic extracts of hemp and milk thistle seed oils showed the highest antiradical activity, whereas the ethanolic extracts revealed the best reducing properties. The obtained antioxidant parameters for hemp seed oil were: the IC_50_ = 3.433 ± 0.017 v/v (DPPH test), the percent of ABTS^•+^ inhibition = 93.301 ± 1.099%, FRAP value = 1063.883 ± 39.225 µmol Fe^2+^, CUPRAC value = 420.471 ± 1.765 µmol of Trolox. Whereas the antioxidant parameters for milk thistle seed oil were: the IC_50_ = 5.280 ± 0.584 v/v (DPPH test), 79.59 ± 3.763% (ABTS test), 2891.08 ± 270.044 µmol Fe^2+^ (FRAP test), 255.48 ± 26.169 µmol of Trolox (CUPRAC assay). Chlorogenic acid effectively inhibited the lipid peroxidation process in hemp and milk thistle seed oils.

## Introduction

Nowadays, there is an increased interest in antioxidants from plant sources. Because of their natural origin they are considered as non-toxic, safe for human and environment as well as biodegradable compared to the synthetic antioxidants. Moreover the extraction of antioxidants from plants does not require the use of large amounts of chemical reagents compared to expensive (and sometimes harmful) organic synthesis. Therefore the application of natural antioxidants is more environmentally friendly and more acceptable by consumers e.g. as food antioxidants, diet supplements or functional food ingredients^1^. Phenolic compounds belong to the main group of plant antioxidants, common components of plant-based diet, which are extensively studied as a chemopreventive compounds as well as potential drugs to treat the free radical-induced diseases, including cancer, diabetes, asthma, neuro-degenerative diseases, and skin aging^2^. The antioxidant defense mechanism of phenolic compounds involves among others the free radical scavenging activity, protection against lipid peroxidation or chelation of toxic metals^3^.

The cold-pressed edible oil market is constantly expanding with new oils extracted from a wide range of plants. Some of the oils that are currently popular in the market are hemp oil, milk thistle oil, evening primrose oil, pumpkin seed oil, black cumin oil, or flaxseed oil. The global cold-pressed oils market is estimated to reach a value of US$ 36.40 billion by 2026, compared to a value of US$ 24.62 billion in 2018. This is attributed to the growing consumer interest in unprocessed food from natural sources, which is rich in antioxidant compounds^4^. Cold-pressed oils, unlike refined oils, retain higher amount of valuable substances in their composition, including phenolic compounds, phytosterols, tocopherols, carotenoids or polyunsaturated fatty acids. Moreover, the method of obtaining oils by cold pressing is simple, does not require high energy inputs and addition of chemicals which makes it an economical and green method. The cold-pressed oils can only be purified by means of water, sedimentation, filtration and centrifugation. On the other hand, the difficulty of this process is obtaining a product of consistent quality. An important factor limiting the wide use of cold pressing is the low efficiency of pressing and the difficulty in obtaining a product with constant parameters and high quality which depends on several factors. The quality of the oils is determined by the type of raw material and its variety, the degree of seed maturity, climatic conditions of cultivation, agrotechnical treatment, transport and storage conditions, grain moisture, cleanliness and no damage of the grains. Moreover, the oxidative stability of cold-pressed oils obtained from seeds is very often lower than their refined counterparts. This can be explained by the removal of pro-oxidizing chemicals during refining (e.g. metals, chlorophyll dyes, hydrolysis and oxidation products) what improves the oxidative stability of refined oils. For this reason, adding selected natural antioxidants to the cold-pressed oils can improve their quality. It should be remembered that the use of cold pressing alone, without further processing—refining, allows the final product to retain many valuable compounds including antioxidants The presence of high content of bioactive compounds in the cold-pressed oils gives them a chance to be called functional food as they have health benefits in addition to their nutritional properties^5^.Hemp (*Cannabis sativa* L.) from the Cannabis family (*Cannabaceae*) is a versatile annual herbaceous plant that has been cultivated for many years mainly in Central Asia. Hemp seeds contain about 20–25% protein, 20–30% carbohydrates, 25–35% oil and 10–15% insoluble fiber, wide range of minerals, nutrients and bioactive compounds including antioxidants, vitamins^6^. Hemp products strengthen the immune system, prevent diabetes, cardiovascular disease, and fight inflammation^7^. The chemical composition of hemp oil, including the high content of essential fatty acids, has led to the name of hemp oil as the best-balanced vegetable oil^8^. Hemp oil is used, among others, to treat atopic dermatitis, psoriasis, osteoporosis, PMS (premenstrual syndrome), menopause, inflammation of the bladder and bowel diseasesor stomach cramps, because its diastolic effect^9^. In addition, hemp seed oil shows weak antibacterial activity against *Bacillus cereus*, *Bacillus subtilis subsp. subtilis*, *Micrococcus luteus*, *Staphylococcus aureus subsp. aureus* (Gram+), *Citrobacter freundii*, *Enterococcus faecalis*, *Escherichia coli*, *Salmonella enterica* (Gram-) tested by the disc diffusion method^10^. Currently, hemp is one of the plant raw materials of great interest due to its versatile applications in the food, cosmetic, pharmaceuticaly, energy, textile, paper industries or as a building bio-material.

Thistle (*Silybum marianum* L.) is an annual or biennial plant that belongs to the Asteraceae family. The plant is native to the Mediterranean region of Europe. Today, thistle has spread to other countries in Europe, Asia, Australia, North America, and South America^11^. Thistle fruits contain 30-35% oil in their composition^12,13^. The extract of milk thistle—silymarin—is a mixture of flavonolignans which is reach in silybin A, silybin B, silydianin, silycristine, isosylibin A, and isosylibin B. Sylibin is the component with high biological activity and accounts for 50-70% of silymarin^14,15^. Thistle is recommended primarily for dyspeptic ailments and liver diseases, including toxin-induced liver damage, cirrhosis, or in an adjunctive therapy for chronic inflammation^16–19^. The milk thistle oil is rich in bioactive compounds, such as phenolic acids, tocopherols, fatty acids and phytosterols^20,21^. The extracts from seed oil effectively prevent oxidative stress and restore normal levels of cholesterol, triglycerides, low-density lipoproteins, and liver markers associated with liver pathologies^21,22^. The thistle seed oil is a natural source of vitamin E and is often recommended as a beneficial edible oil^17^. Therefore it is used as an ingredient of dietary supplements, medicines, and cosmetics. Importantly, the health-promoting properties of oils depend on their chemical composition (e.g. content of fatty acids, phenolic compounds), which may vary from year to year and strongly dependents on plant variety, cultivation method, climate conditions and storage method^23^.

Study on the antioxidant activity and phenolic content of unrefined hemp seed oil are limited (Table 1). Most of the research on the composition of oils concerns the content of fatty acids. Hemp oil, which is a relatively new oil available in the market arouses interest among consumers, therefore intensive research on the chemical composition, antioxidant potential and health effect is necessary. Siger et al.^24^ studied the phenolic compound content and antioxidant activity of cold-pressed vegetable oils including hemp seed oil, soybean oil, canola oil, corn oil, grape seed oil, flax seed oil, pumpkin seed oil and rice bran oil. Among the examined oils the hemp oil showed the highest antioxidant activity determined by the DPPH^•^ radical method and showed the highest total content of phenolic compounds determined with Folin-Ciocalteu reagent^24^. Ramadan et al.^25^ described the effect of adding the DPPH^•^ radicals to vegetable oils, obtaining the following order of scavengers of this radical: coriander oil > cumin oil > cottonseed oil > peanut oil > sunflower oil > walnut oil > hemp oil > flaxseed oil > olive oil > nigrum seed oil^25^. In other studies, Yu et al.^26^ determined the total phenolic compound content in cold-pressed hemp seed oil to be 0.44 mg/g as gallic acid equivalents (GAE mg/g). Furthermore, they established the Fe^2^^+^ chelating, antiradical activity against DPPH^•^ radicals and ABTS^•+^ cation radicals as well as antioxidant potential in the ORAC assay. Callaway et al.^27^ reported that hemp oil has higher content of PUFA acids (82%) compared to olive oil (7%)^27^. Grajzer et al.^28^ reported content of PUFA acids in pumpkin oil (50.7%), milk thistle oil (58.4%), camelina oil (66.6%), flaxseed oil (68.8%), walnut oil (70.3%) and rosehip oil (74.6%)^28^. Milk thistle oil is a little-known oil yet (Table 2). In most of the publications the cold-pressed oil were produced in Asian and African countries. From the literature data, we can obtain information on milk thistle oil, e.g. acidity (0.64%), peroxide number (0.34 meq O_2_ kg of oil) obtained by the standard thinning method,the oxidative stability index, determined by the Rancimate method(55.7 h at 80°C and 12.9 h at 100°C),the contents of fatty acids (19.5 SAT, 22.9 MUFA and 57.6% PUFA), phytosterol (2520 mg/kg) and squalene (9.35 mg/kg) determined by gas-liquid chromatography (GLC),alpha-tocopherol content (237.4 mg/kg), total phenolics (1.16 GAE mg/g oil) determined by high-performance liquid chromatography (HPLC). The milk thistle oil possessed the scavenging capacity of the DPPH^•^ radicals (IC_50_ = 3.34 mg/mL) and revealed the total antioxidant capacity (2.29 GAE mmol/L)^19^. The main fatty acids in milk thistle oil were linoleic acid (C18: 2) (57.0–60.3%) and oleic acid (C18: 1) (15.5–22.4%). Moreover, milk thistle oil was rich in α-tocopherol, and the phenolic content was 1.59–4.73 GAE mg/g, and three phenolic acids were identified (vanillic acid, *p*-coumaric acid and silybine). The above results indicate that milk thistle oil can be a source of natural antioxidants and is rich in PUFA and MUFA, and that the chemical composition of the oil changes with the change of the place of cultivation^17,19^.

**Table 1:** Biological properties of hemp seed oil.

Antioxidant/method; parameter
ORAC; 28.2 ± 6.19 (µmol TE/g)^26^
ABTS; 11.4 ± 2.08 (µmol TE/g)^26^, IC_50_ = 99.96 (µg/mL)^29^, IC_50_ = 66.6 (µg/mL)^29^
Chelating properties estimated by 2,2-bipyridyl reagent/assay; 10.5 ± 0.83 (EDTA mg/g)^26^
TCA; 0.44 ± 0.01(mg GAE/g)^26^, 2.45 ± 0.05 (mg CAE/100 g)^24^
DPPH; IC_50_ = 10.7 mg/mL, IC_50_ = 9.2 mg/mL^29^, EC_50_ = 8,7 µg^24^
**Antimicrobial**
Antibacterial activity against *Bacillus cereus* (Inhibition Zone Size 2.3 ± 0.6 mm), *Bacillus subtilis subsp. Subtilis* (2.3 ± 1.8 mm), *Micrococcus luteus* (3.3 ± 1.8 mm), *Staphylococcus aureus subsp. aureus* (3.0 ± 0.0 mm), *Citrobacter freundii* (2.3 ± 0.6 mm), *Enterococcus faecalis* (2.3 ± 0.6 mm), *Escherichia coli* (0.3 ± 0.6 mm), *Salmonella enterica* (3.0 ± 1.8 mm)^10^
Antifungal activity against *Microsporum canis*^30^
**Anti-diabetes**
Protection against atherosclerosis, reduce the level of LDL, increase the level of HDL^31^
Stimulation of blood platelet aggregation^32^
**Anti-inflammatory**
Reduction of bladder and bowel diseases^9^
Hemp seed extract rich in lignanamides (LnHS) significantly inhibits U-87 cancer cell proliferation^29^

*TE* trolox equivalent, *TCA* total phenolic/antioxidant content, *CAE* caffeic acid equivalents, *EDTA* equivalent represents the chelating capacity, *LDL* low-density lipoprotein, *HDL* high-density lipoprotein, *FRAP* ferric ion reducing antioxidant parameter

**Table 2:** Biological properties of milk thistle seed oil.

Antioxidant/method; parameter
ABTS; 70.25—90.35%^33^
TCA; 1.16 mg (mg GAE/g)^19^
DPPH; IC_50_ = 3.35 mg/mL^19^, %inh = 96.42%^33^
FRAP; 122.14—211.06 M/mL^13^
**Anti-diabetes**^34^
**Anti-inflammatory effect**^34^
**Hepatoprotective properties**^34^
**Antiproliferative effect**^13^
**Anti-arthritic potential**^33^

The aim of this study was to perform the quantitative and qualitative analysis of chemical composition (including phenolic compounds, fatty acids) and antioxidant activity of virgin, unrefined and cold-pressed oils from hemp and milk thistle seeds manufactured in Poland and provided by the oil manufacturer. Four different spectrophotometric antioxidant assays were employed: DPPH^•^ (with the use of (2,2-diphenyl-1-picrylhydrazayl) radical), ABTS^•+^ (with the use of 2,2'-azino-bis(3-ethylbenzothiazoline-6-sulfonic acid) cation radical) as well as FRAP (ferric ion reducing antioxidant parameter) and CUPRAC (cupric ion reducing antioxidant capacity) assays. To this aim the oils were extracted by the use of methanol and ethanol by means of simple and solid phase extraction (SPE). The best way to extract antioxidants from oils was discussed.

## Materials and methods

### Materials

The cold-pressed, virgin and unrefined oils from hemp and milk thistle seeds were obtained from Olejowe Smaki company (north-east Poland, Podlaskie Voivodeship, Bialystok). The seeds used to produce the oils came from Polish plantations. The use of plant materials in the present study complies with the international, national, and institutional guidelines and legislation. The seeds were gently crushed and then pressed in a screw press at a temperature not exceeding 40 ° C. The seeds were crushed only once. The oils have only been cleaned by sedimentation, not exposed to high temperatures, chemicals or water. No preservatives, additives or dyes were added. Methanol (CH_3_OH), ethanol (C_2_H_5_OH), n-hexane (C_6_H_14_), Folin-Ciocalteu reagent, deionized H_2_O, sodium carbonate (Na_2_CO_3_), acetate buffer, phosphate buffer pH=7, iron(III) chloride (FeCl_3_·6H_2_O), iron(II) chloride (FeCl_2_), copper(II) chloride (CuCl_2_), neocuproine (2,9-dimethyl-1,10-phenanthroline), ammonium acetate (C_2_H_7_NO_2_), ammonium thiocyanate, potassium persulfate (K_2_S_2_O_8_), Tween 20 (C_58_H_114_O_26_), TPTZ (2,4,6-tripyridyl-s-triazine), DPPH (2,2-diphenyl-1-picrylhydrazyl), ABTS (2,2-azino-bis(3-ethylbenzothiazoline-6-sulfonic acid), hydrochloric acid 37% (HCl), acetic acid (CH_3_COOH), chlorogenic acid, Trolox (6-hydroxy-2,5,7,8-tetramethylchroman-2-carboxylic acid), *o*-coumaric acid, *m*-coumaric acid, *p*-coumaric acid, caffeic acid, ferulic acid, isoferulic acid, sinapic acid, chlorogenic acid, quercetin, epicatechin, phloridzin, L-ascorbic acid, BHA (butylated hydroxyanisole), BHT (butylated hydroxytoluene), acetonitrile for HPLC, standards of phenolic compounds: gallic acid, protocatechuic acid, procyanidin B1, 2,5-hydroxybenzoic acid, catechin, chlorogenic acid, vanillic acid, caffeic acid, syringic acid, procyanidin B2, (-)epicatechin, procyanidin C1, p-coumaric acid, ferulic acid, rutin, procyanidin A2, quercetin-3-glucoside, quercetin, kaempferol, phloridzin were bought from Sigma-Aldrich (St. Louis, MO, USA). Chemical reagents were of analytical purity. Additionally, the purity of these compounds was confirm by FT-IR spectroscopy and melting point measurement.

#### Oil extraction

**Figure 1:**
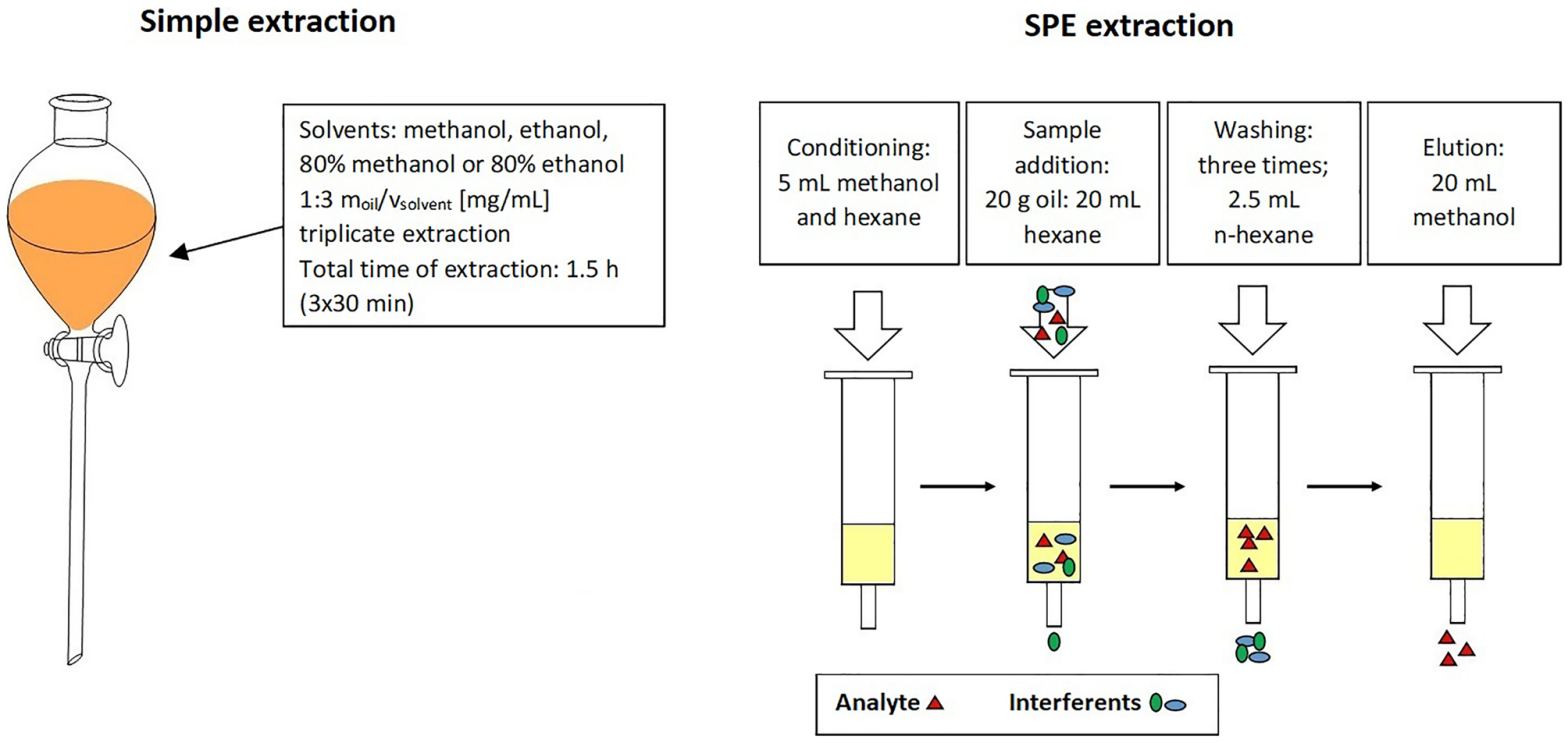
Scheme of hemp and milk thistle seed oils extraction.

In order to obtain oil extracts, the research material was extracted using two extraction techniques: multi-stage classical liquid-liquid extraction and solid phase extraction (Figure 1). Classical liquid-liquid extraction was performed in different solvents, i.e. methanol, ethanol and an aqueous solution of 80% methanol and 80% ethanol. The simple extraction consisted in shaking the weighed mass of oil with the appropriate volume of the solvent for 30 minutes. The total ratio of oil weight to solvent volume during extraction was 1:3 (i.e. 10 g:10 mL). The total extraction time was 1.5 h. At this time, the solution was poured into a separating funnel and allowed to wait for 10 minutes. Then the top layer was poured into a bottle, and the remaining layer was subjected to another extraction. Extraction was performed in triplicate for three independent experiments at room temperature. For solid phase extraction the SPE vacuum manifold Baker 12 positions equipped with Bakerbond SPE C-18 columns was used. First, columns were washed with 5 mL of methanol and then 5 mL of n-hexane. About 20 g of hemp oil was thoroughly mixed with 20 mL of n-hexane and the sample was carefully dosed onto the same columns. The liquid flow rate was controlled using a vacuum pump. Next, the columns were washed three times with 2.5 mL n-hexane to remove the non-polar oil fraction. At the end, the samples were eluted with 20 mL of methanol. The actual extracts were collected in glass bottles and left for further analysis.

#### HPLC analysis

The qualitative and quantitative chromatographic analyses of phenolic compounds present in extracts of hemp and milk thistle seed oils were performed on an Agilent 1260 Infinity liquid chromatograph with a UV spectrophotometric detector, using a Zorbax Eclipse Plus C18 Analytical precolumn column (4.6 x 250 mm; 5 µm). A mixture of acetonitrile and acetic acid with a concentration of 2% and a mobile phase velocity of 1 mL/min was used for the gradient elution, as described in Kalinowska et al.^35^. The autosampler was used for dispensing samples with a sample capacity of 0.01 mL. The chromatographic peaks were recorded at three wavelengths λ= 280, 320, 360 nm. The content of phenolic compounds was determined on the basis of standard curves for solutions of the standard mixture in the concentration range from 5 to 50 mg/L. Samples of extracts for HPLC analysis of known mass (weighed with an accuracy of 0.001 g) were dissolved in 10 mL of methanol, filtered through membrane filters with a pore diameter of 0.45 µm, and then analyzed by liquid chromatography.

#### Physico-chemical properties

The melting point, flash-point, density, total acid number, iodine value and saponification number were established according to the methodology described in^36^.

#### Determination of total antioxidant content (TCA)

TCA was determined using the method described by Singleton and Rosi^37^. The total content of phenolic compounds was expressed as gallic acid equivalent, i.e. mg GAE/g oil. Equation of the standard curve in ethanol: y= 0.008x + 0.0142; R^2^= 0.9941 and methanol: y= 0.0055x + 0.0014; R^2^= 0.9963. In order to determine the standard curves: 5 mg of gallic acid was weighed and dissolved in ethanol using a 25 mL flask. In this way, a solution with a concentration of 200 mg/L was obtained. Nine successive dilutions of gallic acid were made: 100 mg/L; 75 mg/L; 50 mg/L; 25 mg/L; 15 mg/L; 10 mg/L; 7 mg/L; 5 mg/L; 2.5 mg/L. Then the following reagents were mixed: 250 µL of gallic acid solution with a defined concentration; 250 µL of Folin-Ciocalteu reagent diluted 1:1 by volume with deionized water; 500 µL 14% sodium carbonate; 4 mL of deionized water. The test tubes were mixed and incubated for 1 h in dark at room temperature. Then the absorbance of the solutions was measured at the wavelength λ= 760 nm against a blank sample, where the solvent was used instead of gallic acid solution. In order to measure the TCA in oil, 250 µL of oil extract, 250 µL of Folin-Ciocalteu reagent, 500 µL 14% sodium carbonate and4 mL of deionized water were mixed. Samples were incubated for 1 hour at room temperature, protected from light. After this time, the absorbance was measured at the wavelength λ= 760 nm against the blank using UV/VIS/NIR Agilent Carry 5000 spectrophotometer (Santa Clara, CA, USA). The experiment was performed in five replications for three independent experiments.

#### Determination of ferric reducing antioxidant power (FRAP) assay

FRAP activity was determined according to Rice-Evans et al.^38^. The FRAP working solution was prepared in a 10:1:1 volume ratio by mixing 1000 mL of acetic buffer (300 mM), 100 mL of TPTZ (the iron-2,4,6-tris-2-picryl-s-thiazine complex, 10 mM) and 100 mL of FeCl_3_∙6H_2_O solution (20 mM). Then the FeSO_4_∙7H_2_O standard curve was determined. The concentration of the stock solution was 1.5 mM. Serial dilutions of the iron(II) sulfate solution were made: 0.5; 0.45; 0.4; 0.35; 0.3; 0.25; 0.2; 0.15; 0.1; 0.076; 0.05 mM. Then, 0.4 mL of the solution with a given concentration of FeSO_4_ and 3 mL of the FRAP solution were incubate in a dark place at room temperature for 8 minutes. After this time, the absorbance of the solutions was measured at a wavelength of 595 nm against a blank containing 0.4 mL of distilled water and 3 mL of FRAP solution (with the use of UV/VIS/NIR Agilent Carry 5000 spectrophotometer (Santa Clara, CA, USA)). The determination was performed in triplicate. Standard curve equation was: y= 0.0019x–0.0169; R^2^= 0.9973. In order to establish the reducing activity of oils, the samples of the extracts were diluted as follows: 0.1 mL of the oil extract; 0.3 mL of the solvent; 3 mL of FRAP solution. The samples were incubated for 8 min in the dark and then the absorbance was measured at wavelength λ= 595 nm against the blank (0.4 mL solvent and 3 mL FRAP solution). The antioxidant activity was expressed as Fe^2+^ equivalents [μM].

#### Cupric ion reducing antioxidant capacity (CUPRAC) assay

The experiment was conducted according to method de-scribed by Apak et al.^39^. 250 mL of CuCl_2_ (C= 10 mM; aqueous solution) was mixed with 250 mL of ammonium acetate (pH 7; aqueous solution) and neocuproine (C= 75 mM; in ethanol). Then, 3 mL of the CUPRAC mixture was added to 0.5 mL of oil extract and 0.6 mL of distilled water. The samples were vortexed and incubated for 60 min at room temperature (23°C). The absorbance of the samples was measured with the use of UV/VIS/NIR Agilent Carry 5000 spectrophotometer (Santa Clara, CA, USA) at λ= 450 nm against a blank (solvent instead of the sample). Cupric reducing antioxidant activity was estimated using the calibration curve obtained for Trolox (in the concentration range 50–350 µM). Standard curve equation: y= 1.7302x–0.0042; R^2^= 0.9988. Activity was expressed as Trolox equivalents [µM].

#### DPPH assay

Briefly, a 60 µM solution of DPPH^•^ in methanol was prepared. The oil extract, 2 mL DPPH^•^ solution and solvent (methanol or ethanol) were added to the test tube, so that the volume percent concentration of the oil extract in the sample was in the range of 0.66–33.33% [v/v]. The test tubes were mixed using Vortex and kept in the dark for 1 hour. The absorbance at 517 nm was then measured against a blank and the activity was calculated from the formula: I%= [(A_517_ control–A_517_ sample)/A_517_ control] × 100 [%], where A_517_ control—the absorbance of control sample measured at 517 nm, A_517_ sample—the absorbance of test sample measured at 517 nm using UV/VIS/NIR Agilent Carry 5000 spectrophotometer (Santa Clara, CA, USA) against methanol as blank. In the next step, the percent inhibition curves were plotted for the individual oil extracts and the IC_50_ values were calculated^38^.

#### ABTS assay

The antiradical activity of oils was measured by the use of the ABTS method according to^38^. First, 2,2-azino-bis(3-ethylbenzothiazoline-6-sulfonic acid) diammonium salt (ABTS) and potassium persulfate (K_2_S_2_O_8_) were dissolved in distilled water to a final concentration of 7 mM and 2.45 mM, respectively. Then, the solutions were mixed in volume ratio of 1:1 (v/v) and left for 16 h at room temperature (23°C) to produce ABTS cation radical (ABTS^•+^). After that, ABTS^•+^ was diluted with methanol to obtain a solution with an absorbance of ~0.700 at 734 nm. Then, 1 mL of the oil extract was added to 1 mL of the ABTS^•+^ solution, and after 7 min the absorbance was read at 734 nm wavelength against solvent with the use UV/VIS/NIR Agilent Carry 5000 spectrophotometer (Santa Clara, CA, USA). The percentage of inhibition of the ABTS^•+^ cation radicals was estimated according to formula: %I = [(A_734_ control—A7_34_ sample)/A_734_ control]×100 [%], where A_734_ control—the absorbance of control sample measured at 734 nm, A_734_ sample—the absorbance of test sample measured at 734 nm.

#### Fatty acids composition

It was determined according to the method described in^40^.

#### Influence of chlorogenic acid on the lipid peroxidation process

In order to determine the lipid peroxidation, a 30% water solution of ammonium thiocyanate and 0.02 M solution of iron(II) chloride in 3.5% hydrochloric acid were prepared. In order to prepare the appropriate concentration of the solutions, 15 g of NH_4_SCN was weighed and dissolved in 50 mL of distilled water and 0.1988 g of FeCl_2_ in 50 mL of 3.5% HCl. Then a milk thistle or hemp seed oil emulsion was prepared by taking 2 mL of appropriate oil and 2 mL of Tween 20 and diluting with phosphate buffer to a volume of 50 mL. The concentration of chlorogenic acid in the trials was: 4.00, 0.40 and 0.04 mM. The samples consisted of: 1.5 mL of emulsion and 1mL of chlorogenic acid solution with successive concentrations. Samples were prepared in triplicate for each concentration of chlorogenic acid, screwed, mixed and left for incubation at 40°C. After 1 hour, the first measurement was made. For this purpose, 0.1 mL of solution from the incubated samples was taken, and 4.7 mL of 75% methanol and 0.05 mL of a 30% ammonium thiocyanate solution were added to the same test tubes. After 3 minutes the 0.05 mL of a 0.02 M solution of iron(II) chloride in hydrochloric acid was added. The absorbance at 500 nm was read immediately in relation to 75% methanol using UV/VIS/NIR Agilent Carry 5000 spectrophotometer (Santa Clara, CA, USA). Measurements were made through the next 4 days every 24 hours. The degree inhibition of lipid peroxidation was calculated from the formula: %I= [(A_500_ control—A_500_ sample)/A_500_ control]×100 [%], where A_500_ control—the absorbance of control sample measured at 500 nm, A_500_ sample—the absorbance of test sample measured at 500 nm.

All antioxidant assays were performed in five replications for three independent experiments. The results were expressed as the means of the values obtained for the replications. Average, standard deviation calculation, and graphs were completed using Microsoft Excel 2019.

## Results and discussion

### Physico-chemical properties

In Table 3 the selected physico-chemical parameters of studied oils were shown. The total acid number determined for milk thistle seed oil was much higher than for hemp seed oil. Probably, the milk thistle seed oil may contain higher amount of fatty acids. The iodine value was higher for hemp seed oil, probably because of the larger content of unsaturated fatty acid compared with milk thistle seed oil. The saponification number was similar for both oils.

**Table 3:** Selected physico-chemical parameters of milk thistle and hemp seed oils.

Parameter	Milk thistle seed oil	Hemp seed oil
Melting point	−2 °C	−8 °C
Flash-point	> 300 °C	240 °C
Density	0.9140 g/mL	0.9280 g/mL
Total acid number	13.60 mg KOH/g_oil_	2.15 mg KOH/g_oil_
Iodine value	99 mg I_2_/100g_oil_	166 mg I_2_/100g_oil_
Saponification number	195 mg KOH/g_oil_	193 mg KOH/g_oil_

### HPLC analysis

HPLC analysis showed that the content of phenolic compounds in the extracts differs in terms of qualitative and quantitative composition depending on the type of extraction and the solvent. In both cases, the extracts obtained by the simple extraction method showed a higher content of phenolic compounds than the extracts obtained by solid phase extraction (SPE). The total content of phenolic compounds in hemp seed oil varied from 0.303 ± 0.049 μg/g_oil_ to 1.476 ± 0.001 μg/g_oil_. The highest content of phenolic compounds (1.476 ± 0.001 μg/g_oil_) was obtained in the 80% methanolic extract (interestingly, only (-)epicatechin was determined there). On the other hand, the lowest content of phenolic compounds was in the ethanolic extract (0.303 ± 0.049 μg/g_oil_). The use of 80% ethanol and methanol as a solvent made it possible to extract the largest number of various phenolic compounds in terms of the number. Methanolic extract (simple extraction) was abundant in kaempferol, vanillic and ferulic acids. 80% methanol is good solvent for (-)epicatechin extraction from hemp seed oil. Whereas the highest amount of procyanidin B2, catechin and *p*-coumaric acid was obtained in 80% ethanolic extract of hemp oil (Table 4). Siger et al.^24^, who studied the quality characteristics of cold-pressed Polish hemp seed oil, showed the presence of phenolic compounds such as *p*-hydroxybenzoic acid (6.0 ± 0.06 µg/100 g_oil_), vanillic acid (2.0 ± 0.10 µg/100 g_oil_), *p*-coumaric acid (2.0 ± 0.15 µg/100 g_oil_), ferulic acid (1.0 ± 0.08 µg/100 g_oil_) and sinapic acid (3.0 ± 0.05 µg/100 g_oil_)^24^. Moreover, Smeriglio et al.^39^, defined the presence in methanolic extract of cold-pressed Italy hemp seed oil some of phenolic acids, flavanones, flavonols, isoflavones, flavones and flavanols. In other study, the identified compounds in hemp seed oil were vanillic acid, catechin and epicatechin^41^. Only *p*-coumaric acid and vanillic acid have been identified in Tunisian milk thistle oil, and the amount of *p*-coumaric acid ranged from 0.26 mg to 0.9 mg/100 g_oil_. Meddeb et al. found three phenolic compounds in silybine, vanillic and *p*-coumaric acids in Tunisian milk thistle seed oil. Vanillic acid was predominant, its concentration reached 40.2 and 83 mg/100 g_oil_ (depending on the origin of the oil)^17^. In other studies of Tunisian milk thistle seed oil, the following compounds were identified: homovanillic acid (1.30 and 2.10 mg equivalents of quercetin (QE)/kg_oil_), vanillin (2–4.40 mg QE/kg_oil_), *p*-coumaric acid (0.70 and 1 mg QE/kg_oil_), quercetin-3β-glucoside (0.8 and 1 mg QE/kg_oil_), quercetin (1.20–2.50 mg QE/kg_oil_), apigenin (0.9–1.8 mg QE/kg_oil_). The total content of polyphenols in the milk thistle oil was 12.8 mg equivalent quercetin/kg_oil_^12^.

**Table 4:** Chromatographic analyses of phenolic compounds in the of hemp and milk thistle seed oils.

Hemp seed oil										
Phenolic compound	Simple extraction						SPE extraction			
	Methanolic extract		Ethanolic extract		80% Methanolic extract		80% Ethanolic extract		Methanolic extract	
	µg/g_extract_	μg/g_oil_*	µg/g_extract_	μg/g_oil_*	µg/g_extract_	μg/g_oil_*	µg/g_extract_	μg/g_oil_*	µg/g_extract_	μg/g_oil_*
Vanillic acid	0.283 ± 0.002	0.260 ± 0.002	0.172 ± 0.012	0.159 ± 0.011	–	–	–	–	0.042	0.039
Ferulic acid	0.105 ± 0.001	0.097 ± 0.001	–	–	–	–	0.037	0.034	–	–
*p*-coumaric acid	–	–	–	–	–	–	0.058	0.054	–	–
(-)epicatechin	0.702 ± 0.026	0.651 ± 0.024	–	–	1.591 ± 0.001	1.476 ± 0.001	–	–	–	–
Catechin	–	–	–	–	–	–	0.617	0.572	0.241	0.224
Kaempferol	0.168 ± 0.004	0.156 ± 0.003	0.156 ± 0.041	0.144 ± 0.038	–	–	0.039	0.036	0.039	0.036
Procyanidin B2	–	–	–	–	–	–	0.423	0.392	0.291	0.270
Milk thistle seed oil										
Phenolic compound	Simple extraction						SPE extraction			
	Methanolic extract		Ethanolic extract		80% Methanolic extract		80% Ethanolic extract		Methanolic extract	
	µg/g_extract_	μg/g_oil_*	µg/g_extract_	μg/g_oil_*	µg/g_extract_	μg/g_oil_*	µg/g_extract_	μg/g_oil_*	µg/g_extract_	μg/g_oil_*
Protocatechuic acid	–	–	–	–	–	–	0.089 ± 0.009	0.081 ± 0.008	–	–
Catechin	0.834 ± 0.036	0.762 ± 0.032	–	–	–	–	–	–	–	–
Syringic acid	–	–	–	–	–	–	–	–	0.040	0.037
Procyanidin B2	–	–	0.642 ± 0.045	0.587 ± 0.081	0.619 ± 0.039	0.566 ± 0.035	–	–	–	–
Procyanidin C1	0.612 ± 0.180	0.559 ± 0.164	–	–	0.902 ± 0.024	0.825 ± 0.022	–	–	–	–
*p*-coumaric acid	0.032 ± 0.004	0.029 ± 0.003	–	–	0.080 ± 0.001	0.073 ± 0.001	0.090 ± 0.001	0.080 ± 0.001	–	–
Phloridzin	0.940 ± 0.091	0.859 ± 0.083	0.895 ± 0.088	0.818 ± 0.081	1.755 ± 0.007	1.604 ± 0.006	1.618 ± 0.009	1.479 ± 0.008	1.362	1.245
Quercetin	0.075 ± 0.003	0.069 ± 0.003	0.082 ± 0.009	0.075 ± 0.008	0.159 ± 0.001	0.149 ± 0.001	0.243 ± 0.008	0.222 ± 0.007	0.172	0.156
Kaempferol	–	–	–	–	0.059 ± 0.001	0.054 ± 0.001	0.123 ± 0.054	0.112 ± 0.050	0.062	0.057

**Figure 2:**
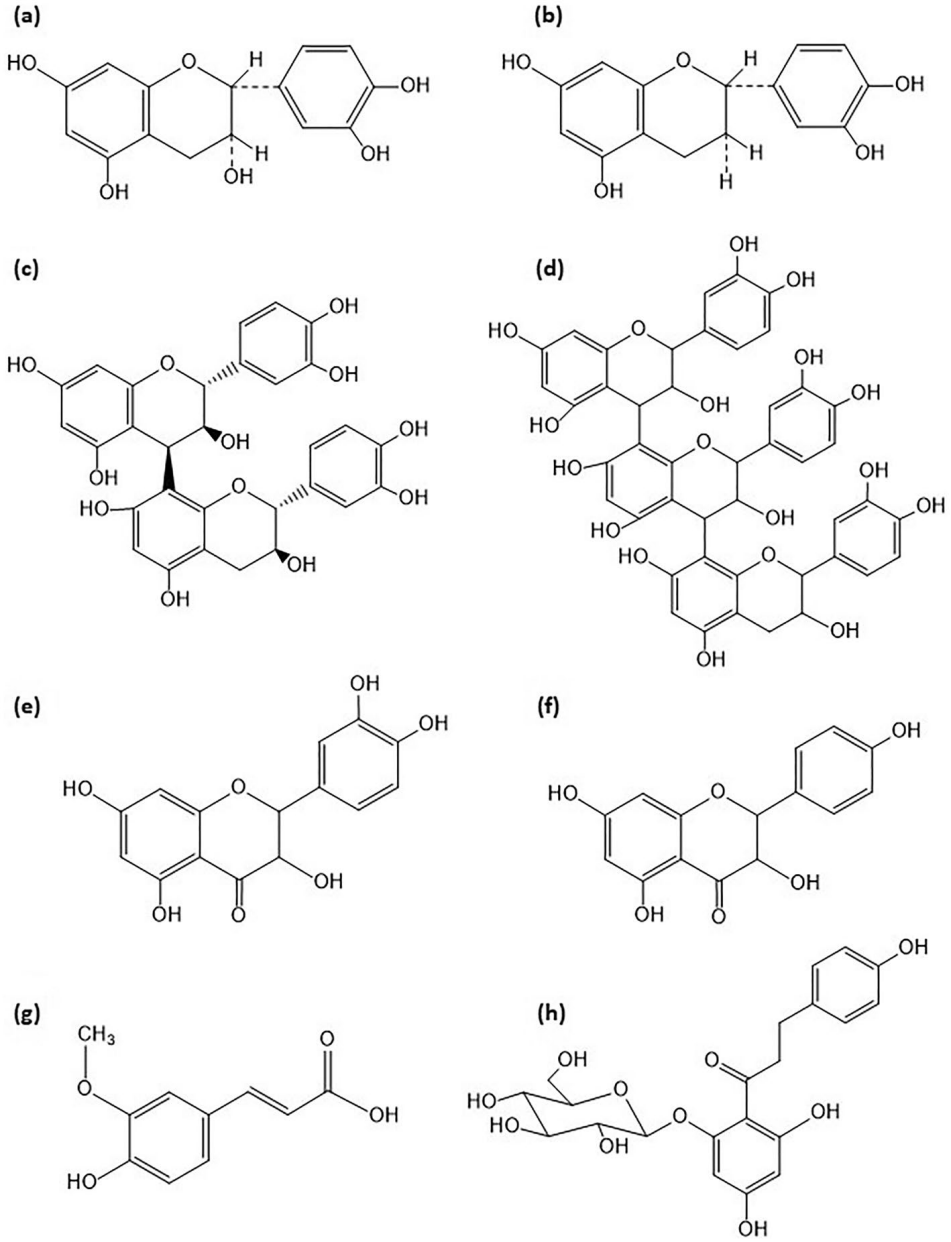
The structure of phenolic compounds found in hemp and milk thistle seed oils: (**a**) (-)epicatechin, (**b**) catechin, (**c**) procyanidin B2, (**d**) procyanidin C1, (**e**) quercetin, (**f**) kaempferol, (**g**) ferulic acid, (**h**) phloridzin.

In our study, the total content of phenolic compounds in milk thistle seed oil ranged from 1.458 μg/g_oil_ to 3.271 ± 0.070 μg/g_oil_. The highest content of phenolic compounds (3.271 ± 0.070 μg/g_oil_) was obtained in the 80% methanolic extract. More phenolic compounds were identified in the thistle seed oil extracts compared with the hemp seed oil extracts. The milk thistle seed oil extracts were abundant in phloridzin. In the extract obtained by simple extraction with the use of 80% methanol the content of phloridzin was on the level of 1.755 ± 0.009 μg/g of extract, which constituted ~49% of the total content of phenolic compounds in the extract. The SPE technique gave the extract with 1.362 μg phloridzin per gram of extract, which was approx. 80% of the total phenolic compounds in this extract. The milk thistle seed oil was more abundant in catechin, procyanidins B2 and C2 compared with hemp seed oil. The literature data suggests that phenolic compounds identified in hemp and milk thistle seed oils (Figure 2) have health-promoting properties. The (-)epicatechin found in hemp oil may prevent oxidative damage, hypertension-related conditions, as well as exhibit anti-cancer effects^42^. Ferulic acid has anti-inflammatory, antioxidant, antibacterial, anticancer and antidiabetic properties. It accelerates wound healing and protects skin structures such as keratinocytes. It is used in cosmetic industry^43^. Catechin activates antioxidant substances, i.e., glutathione, regulates the proliferation of immune cells, and may support the treatment of inflammatory bowel disease^44^.

### Spectrophotometric analysis of the total content of antioxidants (TCA) and antioxidant activity of oils

**Figure 3:**
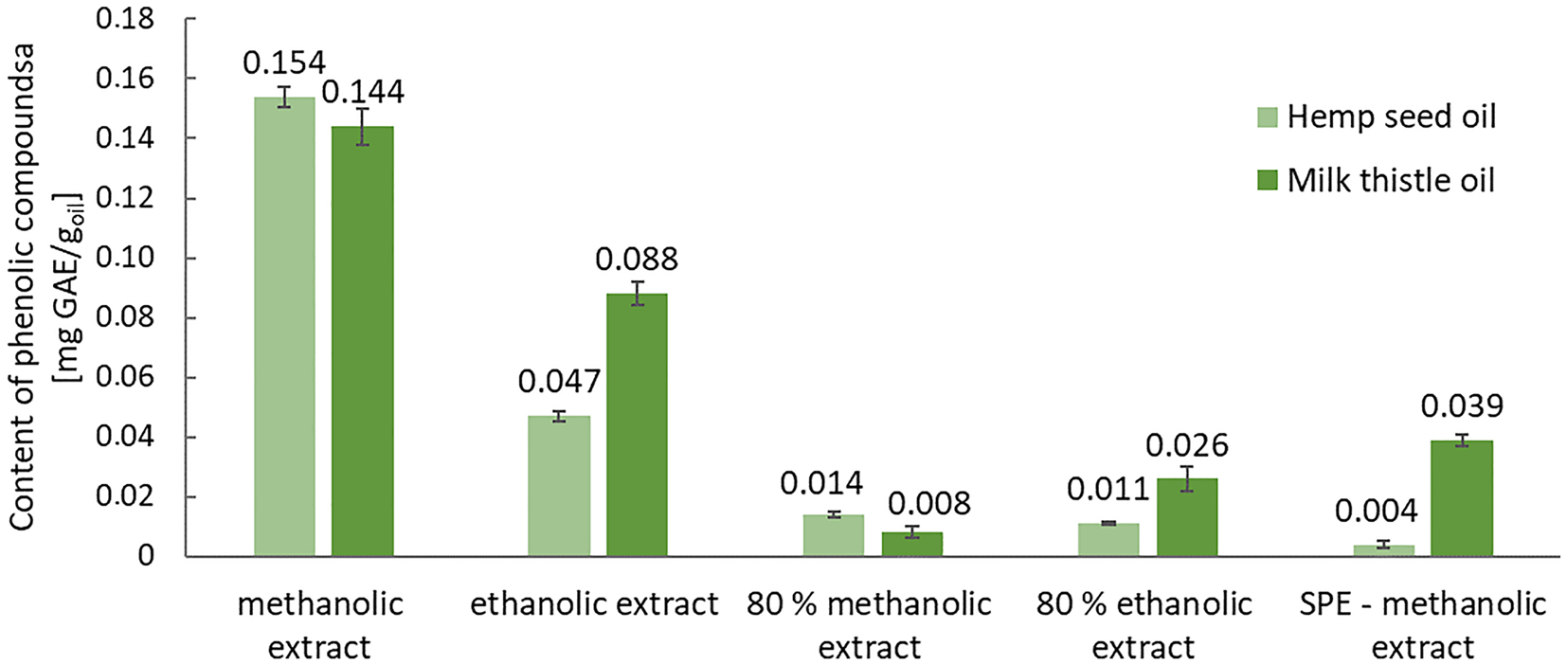
The content of phenolic compounds [mg GAE/g_oil_] depending on the type of extraction and solvent (± standard deviation are depicted).

The total content of phenolic compounds in oils, expressed as gallic acid equivalents per g of oil, was depicted in Figure 3. Depending on the solvent and the extraction technique, the total content of phenolic compounds in the hemp seed oil increased as follows: methanolic extract (SPE, 0.004 ± 0.0010 mg GAE/g_oil_) < 80% ethanolic extract (0.011 ± 0.0004 mg GAE/g_oil_), 80% methanolic extract (0.014 ± 0.0008 mg GAE/g_oil_) < ethanolic extract (0.047 ± 0.0018 mg GAE/g_oil_) < methanolic extract (0.154 ± 0.0035 mg GAE/g_oil_). In the case of milk thistle oil: 80% methanolic extract (0.008 ± 0.002 mg GAE/g_oil_) < 80% ethanolic extract (0.026 ± 0.004 mg GAE/g_oil_) < methanolic extract SPE (0.039 ± 0.002 mg GAE/g_oil_) < ethanolic extract (0.088 ± 0.004 mg GAE/g_oil_) < methanolic extract (0.144 ± 0.006 mg GAE/g_oil_). However, in both oils, the highest total content of phenolic compounds was determined in the methanolic and ethanolic extracts obtained by the simple extraction technique. Only in the case of ethanolic, 80% ethanolic and SPE-methanolic extracts the higher content of phenolic compounds in milk thistle seed oil was estimated by means of Folin-Ciocalteu reagent. It was not the first time where the result of the spectrophotometric assay with Folin-Ciocalteu reagent did not agree with the content of phenolic compounds established by HPLC method^35^. Non-phenolic compounds such as some vitamins and their derivatives (L-ascorbic acid, folic acid, folinic acid, retinoic acid, thiamine), nucleotide bases (e.g. guanine), amino acids (tyrosine, tryptophan, cysteine) and simple inorganic ions (Fe^2﻿+^, Mn^2+^, I^−^ and SO_3_^2−^) react as well with the Folin-Ciocalteu reagent^45^. Nevertheless the assay is commonly applied for the study of the total content of phenolic compounds in food products.

According to the literature data the total content of polyphenols in cold-pressed hemp seed oil from Finola and Fedora cultivar ranged from 0.02 to 2.67 mg GAE/100g_oil_^46^. For comparison, the total phenolic content determined for other oils was 1.48 mg CAE (caffeic acid equivalents)/100g in soybean oil, 1.31 mg CAE/100g in canola oil, 1.20 mg CAE/100g in sunflower oil, 1.26 mg CAE/100g in corn oil, and 1.48 mg CAE/100g in pumpkin oil^24^, which is much lower total TCA compared to studied in this work milk thistle and hemp seed oils In other study, cold-pressed sunflower oil (192.8 mg GAE/100g_oil_) and rapeseed oil (155.9 mg GAE/100g_oil_), extra virgin olive oil (342.1 mg GAE/100g_oil_) possessed higher TCA than studied herein plant oils^47^.

The antioxidant activity of oil extracts can be carried out by several tests that rely on different antioxidant mechanism, e.g. DPPH, ABTS, FRAP, CUPRAC tests. The DPPH and ABTS methods determine the antiradical activity based on the mixed HAT (hydrogen atom transfer) and SET (single electron transfer) mechanisms. According to the both tests, the ability of hydrophilic or lipophilic antioxidants from the oils to reduce the stable radical DPPH^•^ and the cation radical ABTS^•+^ is measured spectrophotometrically. The reducing activity of oil measured in FRAP (ferric ion reducing antioxidant parameter) and CUPRAC (cupric ion reducing antioxidant capacity) test rely on SET mechanism. These tests base on the ability of the antioxidant to reduce iron Fe(III) or Cu(II) ions. It is important to use different tests to study the antioxidant capacity of compounds due to their different antioxidant mechanism which depends on the type of radical, pH value, type of solvent, time of measurement, redox potential. The result of the antioxidant assays were gathered in Table 5.

**Table 5:** The antioxidant activity of hemp and milk thistle seed oils determined by means of DPPH, ABTS, FRAP, CUPRAC assays (method/units); the different ways of oil sample extraction were taken into account is.

Oil	Simple extraction			SPE extraction	
	Methanol	Ethanol	80% Methanol	80% Ethanol	Methanol
**DPPH/IC**_**50**_** [V**_**extract**_**/V**_**mixture**_**]**					
Hemp seed oil	3.43 ± 0.02	3.39 ± 0.04	-	31.55 ± 0.60	-
Milk thistle seed oil	5.28 ± 0.58	5.61 ± 0.74	-	28.96 ± 0.48	-
**ABTS/% inhibition of the ABTS**^**•+**^** radical cation**					
Hemp seed oil	93.30 ± 1.10	90.12 ± 3.59	66.72 ± 1.02	61.65 ± 7.97	21.79 ± 4.96
Milk thistle seed oil	79.59 ± 3.76	25.03 ± 4.24	44.72 ± 8.44	52.90 ± 2.09	67.19 ± 5.31
**CUPRAC/CUPRAC units [µM**_**Trolox**_** /L **_**extract**_**]**					
Hemp seed oil	132.56 ± 10.94	420.47 ± 5.86	98.71 ± 3.14	116.35 ± 1.77	56.47 ± 10.32
Milk thistle seed oil	242.57 ± 28.04	255.48 ± 26.17	90.37 ± 8.74	128.32 ± 8.16	45.43 ± 7.00
**FRAP/FRAP units [µM Fe**^**2+**^** /L **_**extract**_**]**					
Hemp seed oil	975.46 ± 90.58	1063.88 ± 39.23	487.04 ± 2.39	338.04 ± 18.35	318.53 ± 63.05
Milk thistle seed oil	873.44 ± 40.75	2891.08 ± 270.04	75.52 ± 4.93	558.60 ± 13.84	107.88 ± 1.24

**Figure 4:**
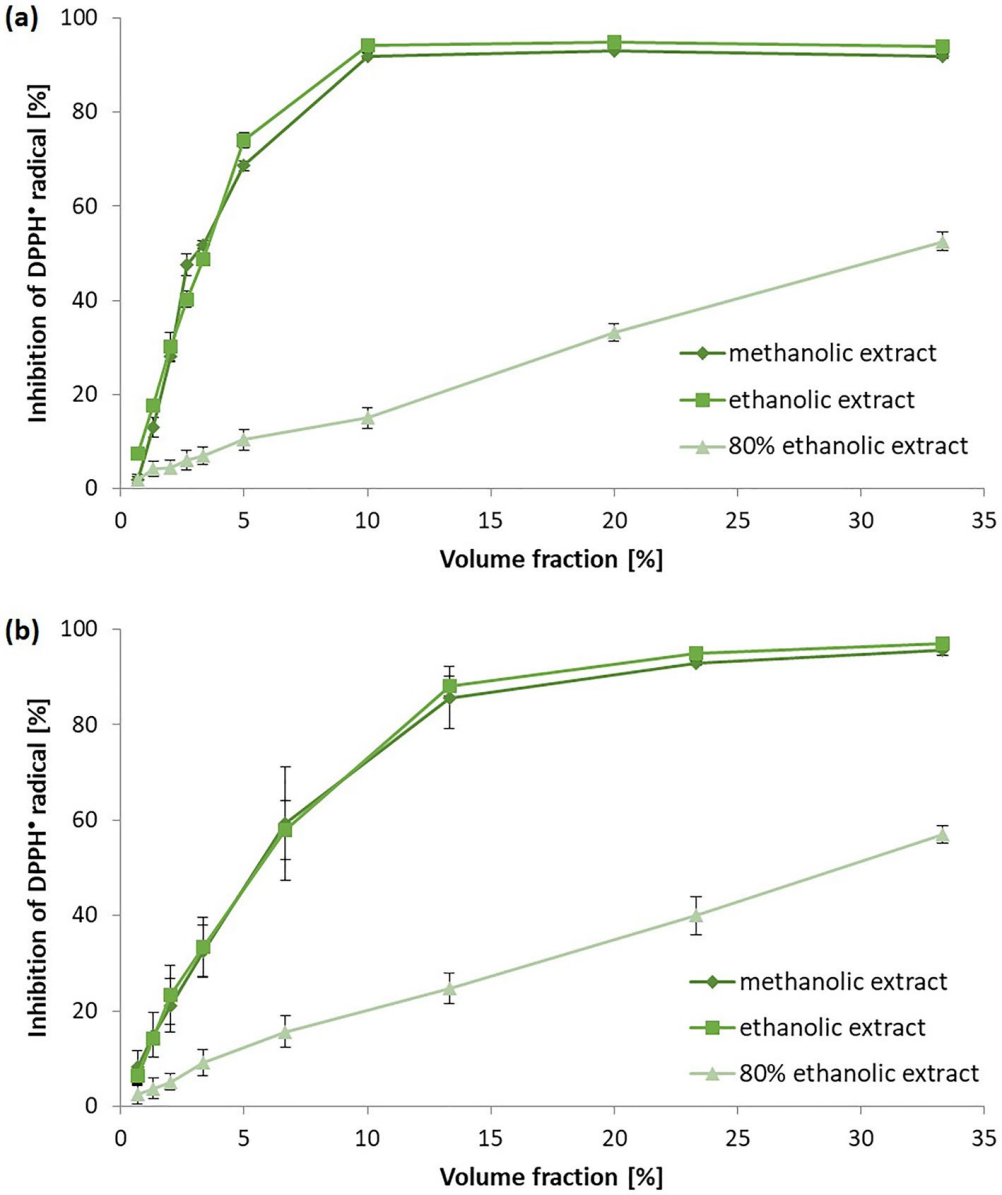
The DPPH^•^ scavenging curve obtained for (**a**) hemp seed oil extracts, (**b**) milk thistle seed oil extracts (methanolic, ethanolic and 80% ethanolic; ± standard deviation are depicted).

Hemp seed oil (IC_50_= 3.43 ± 0.02 v/v methanolic extract; IC_50_= 3.39 ± 0.04 v/v ethanolic extract) showed higher antioxidant activity in DPPH assay than milk thistle seed oil (IC_50_= 5.28 ± 0.58 v/v methanolic extract; IC_50_= 5.61 ± 0.74 v/v ethanolic extract) (Figure 4). The antiradical activity of 80% methanolic and a SPE extracts showed too low DPPH^•^ antiradical activity to be measured in this assays. The hemp seed oil (%I= 93.30 ± 1.10% methanolic extract; %I= 90.12 ± 3.59% ethanolic extract) revealed also higher activity against ABTS^•+^ compared to milk thistle seed oil (%I= 79.59 ± 3.76% methanolic extract; %I= 67.19 ± 5.32% SPE methanolic extract). Generally, the methanolic extracts from the studied oils showed higher antiradical activity (in DPPH and ABTS assays) compared to the ethanolic and the other extracts. This is in accordance with the results obtained by the use of HPLC and spectrophotometric Folin-Ciocalteu analysis. The FRAP and CUPRAC assays showed that the both oils had the iron(III) and copper(II) reducing capacity. In this case the ethanolic extracts of the oils possessed higher reducing properties than the methanolic ones. Generally, the antioxidant activity of plant extracts correlates with the phenolic compound content^48^. But it should be bare in minds that not only the total amount of phenolics, but also other parameters including rate of reaction with radicals, pH of reaction, type of solvent, lipophilic character of antioxidant, redox potential, stability of phenoxyl radicals affect the antioxidant capacity of plant extract or chemicals as well.

### Antioxidant activity of selected phenolic compounds from plant oils

**Figure 5:**
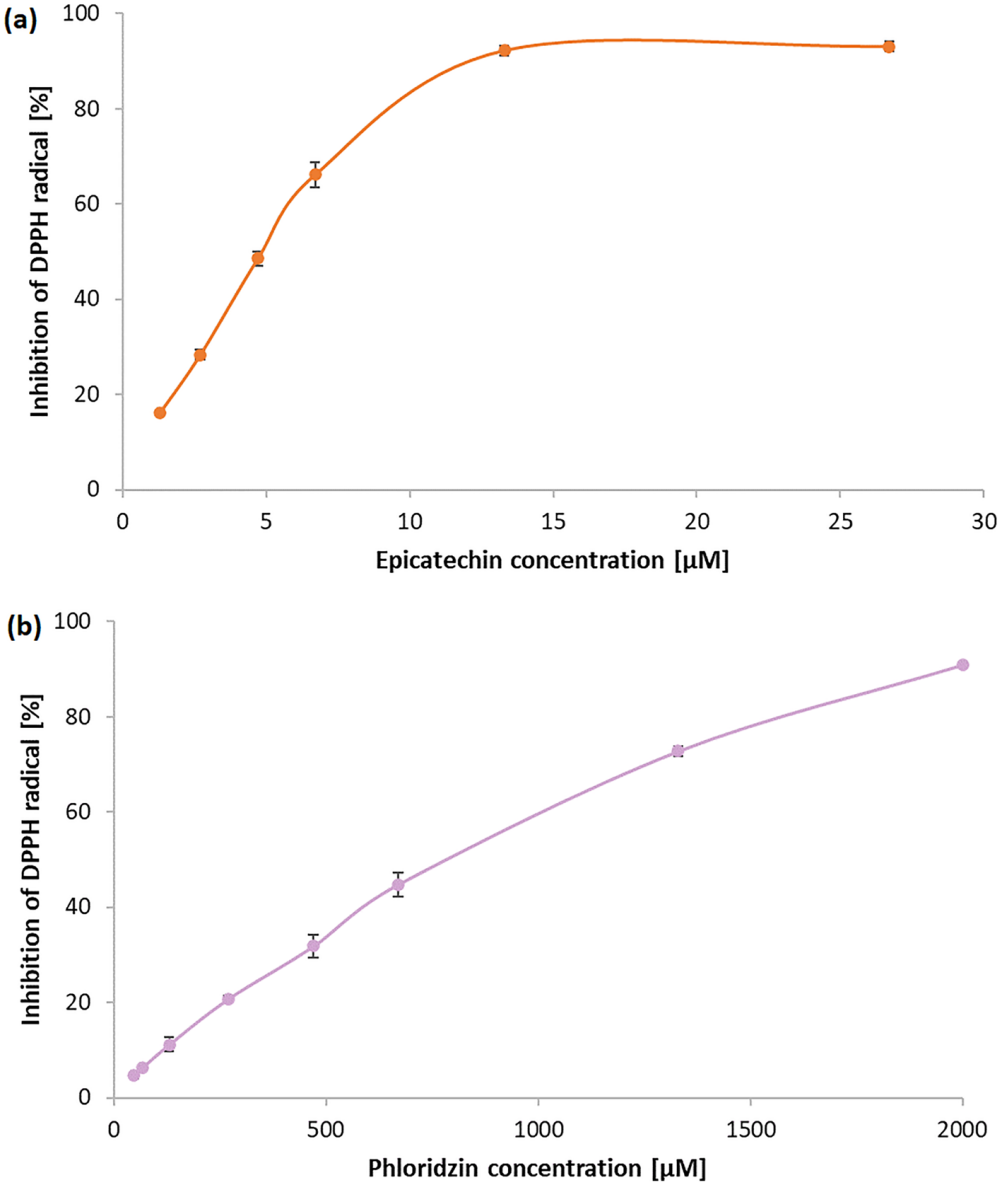
The DPPH^•^ scavenging curve obtained for (**a**) epicatechin (IC_50_ = 4.9 ± 0.2 μM), (**b**) phloridzin (IC_50_ = 837.6 ± 29.4 μM) (± standard deviation are depicted).

The antioxidant activity of selected individual phenolic compounds (including phenolics found in hemp and milk thistle seed oils)was measured by DPPH, FRAP, CUPRAC, and ABTS tests. We chose on purpose the series of hydroxy- and methoxycinnamic acids and their derivatives, as well as natural (L-ascorbic acid) and synthetic antioxidants (BHA, BHT) applied in food industry to check whether the natural products are better antioxidants compared with the synthetic ones and to study the dependency between chemical structure of phenolics and their antioxidant potential. The studied compounds were commercially purchased chemicals (details are included in the Materials and methods section). The obtained results were gathered in Table 6, and illustrated in Figure 5. Among the tested compounds, caffeic acid (IC_50_= 3.29 ± 0.29 µM), quercetin (IC_50_= 3.90 ± 0.01 µM), and epicatechin (IC_50_= 4.90 ± 0.20 µM) (Figure 5a) possessed the highest DPPH^•^ radical scavenging activity (better than commercially applied antioxidants). The *o*-, *m*-, and *p*-coumaric acids showed the lowest antiradical activity (IC_50_ > 13360.55 µM). Phloridzin, which was found to be the major phenolic compound in milk thistle seed oil extracts, showed lower DPPH• radical scavenging properties (IC_50_ = 837.6 ± 29.40 µM) compared to caffeic acid or quercetin (Figure 5b). According to these results, the total antioxidant activity determined in DPPH assay follows the order: *m*-coumaric acid < *o*-coumaric acid < *p*-coumaric acid < phloridzin < isoferulic acid < BHT < ferulic acid < BHA < L-ascorbic acid < sinapic acid ≤ chlorogenic acid < epicatechin < quercetin ≤ caffeic acid. The dihydroxy- and hydroxy, methoxy-cinnamic acids possessed higher antioxidant activity measured in DPHH, ABTS, CUPRAC and FRAP assays compared to monohydroxycinnamic acids. The cinnamic acid substituted in the aromatic ring with two hydroxy groups (i.e. caffeic acid) had higher antioxidant capacity compared to cinnamic acids substituted by hydroxy and methoxy (–CH_3_O) group (i.e. sinapic and ferulic acids). Moreover sinapic acid which aromatic ring is substituted by two methoxy and one hydroxy group occurred better antioxidant compared to ferulic acid with one methoxy and one hydroxy group in the aromatic ring. The same trend was also reported in the study of Tan and Shahidi^49^, Karamać et al.^50^, and Kikuzaki et al.^51^. In the work of Shimoji et al.^52^, sinapic acid isolated from Kurosu (unpolished rice vinegar) also showed higher antiradical activity than ferulic acid (IC_50_= 77.2 and 113.9 µM, respectively). The presence of two hydroxyl (–OH) groups in the molecular structure of caffeic acid affects its higher antioxidant activity compared to the rest of the compounds from the above series. Phenolic compounds with a large number of –OH groups in their structures, especially 3’,4’-o-dihydroxy- groups (such as caffeic acid, chlorogenic acid, ellagic acid) usually show greater efficacy in protecting LDL (low-density lipoprotein) from oxidation^53^. Sinapic acid has one more methoxy group (in the ortho- position) than ferulic acid. This group increases the electron transfer capacity, which increases the stability of the phenoxyl radical and its antioxidant activity^54^. Due to the presence of the –CH_3_O group placed in the position *ortho*- to the -OH group, ferulic acid shows stronger antioxidant properties than *p*-coumaric acid^55,56^. The results of our DPPH assay have shown that *p*-coumaric acid has a greater antiradical activity that *o*-coumaric acid. The studies carried out by Karamać et al.^50^, and Strazisar et al.^57^ showed the following increase in the inhibition of the DPPH^•^ radicals: *m*-coumaric acid < *o*-coumaric acid < *p*-coumaric acid, which confirms the obtained in our work relationship. *Ortho*- and *para*- isomers, due to the electron-donating effect of the COOH-CH=CH- group on the aromatic ring, show resonance stabilization of the phenoxyl radicals, and thus have a higher antioxidant activity than meta- isomers^57,58^. Among all tested compounds, quercetin possessed the strongest FRAP (566.23 ± 19.47 µM Fe^2+^) and CUPRAC (174.99 ± 2.73 of Trolox equivalents) antioxidant capacity. This activity is closely related to the molecular structure of this flavonoid, in particular to the presence of -OH groups in the 5 and 7 positions of the B ring, which are responsible for the reducing properties of quercetin (the ability to scavenge radicals)^59^. Significant reducing activity of Fe(III) ions was also demonstrated by epicatechin (267.80 ± 8.70 µM Fe^2+^), sinapic acid (254.02 ± 20.74 µM Fe^2+^), caffeic acid (243.70 ± 4.80 µM Fe^2+^), and chlorogenic acid (216.09 ± 4.15 µM Fe^2+^). According to these results, the total antioxidant capacity determined in FRAP assay follows the order: *m*-coumaric acid ≤ *o*-coumaric acid ≤ phloridzin (40.95 ± 6.29 µM Fe^2+^) ≤ *p*-coumaric acid < BHT ≤ BHA < L-ascorbic acid ≤ isoferulic acid < ferulic acid < chlorogenic acid < caffeic acid < sinapic acid ≤ epicatechin < quercetin. L-ascorbic acid and studied isomers of coumaric acid showed the lowest antioxidant properties in the CUPRAC assay. The cuprac reducing antioxidant power of the studied substances can be arranged in the following series: L-ascorbic acid < *p*-coumaric acid ≤ *m*-coumaric acid ≤ *o*-coumaric acid ≤ BHT ≤ ferulic acid ≤ isoferulic acid ≤ BHA < sinapic acid < caffeic acid < chlorogenic acid < quercetin. The results of the ABTS assay demonstrated that all tested compounds showed significant scavenging activity against ABTS^•+^. The percentage of inhibition of ABTS^•+^ cation radicals ranged from 51.93 to 91.44%.

**Table 6:** The antioxidant activity of chosen phenolic compounds determined by means of DPPH, ABTS, FRAP, and CUPRAC assays (* the concentration of tested substances in the samples was 50 µM).

Compound	DPPH/IC_50_ [µM]	FRAP* [µM Fe^2+^]	CUPRAC* /C_Trolox_ [µM]	ABTS* I%
*o*-Coumaric acid	21,514.60 ± 938.47	38.44 ± 3.47	71.50 ± 12.87	67.99 ± 2.66
*m*-Coumaric acid	> 50,000	35.89 ± 3.67	61.32 ± 8.32	51.93 ± 4.30
*p*-Coumaric acid *	13,360.55 ± 725.98	42.44 ± 5.24	52.02 ± 9.96	65.36 ± 3.67
Caffeic acid	3.29 ± 0.29	243.70 ± 4.80	125.07 ± 1.48	84.75 ± 2.58
Ferulic acid *	15.20 ± 4.61	195.92 ± 22.10	79.77 ± 8.39	86.02 ± 1.85
Isoferulic acid	365.27 ± 24.96	159.92 ± 4.44	81.41 ± 5.37	84.73 ± 1.70
Sinapic acid	8.75 ± 0.78	254.02 ± 20.74	94.32 ± 1.43	91.44 ± 2.44
Chlorogenic acid	7.39 ± 0.71	216.09 ± 4.15	129.58 ± 9.70	89.53 ± 3.61
Quercetin *	3.90 ± 0.01	566.23 ± 19.47	174.99 ± 2.73	86.48 ± 1.65
L-ascorbic acid	10.87 ± 0.53	156.86 ± 2.02	25.57 ± 3.50	86.76 ± 1.58
BHA	13.32 ± 1.14	141.82 ± 3.58	83.55 ± 14.97	88.38 ± 2.98
BHT	52.80 ± 2.83	134.40 ± 2.69	74.81 ± 7.57	62.66 ± 5.65

*The phenolic compounds found in the hemp and milk thistle seed oils.

### Fatty acids analysis

Milk thistle seed oil contained higher amount of saturated fatty acids (17–20%) compared with hemp seed oil (9–10%) (Table 7). Lauryl and myristic acids were not determined in hemp seed oil, whereas the content of palmitic and arachidic oils was halved compared to milk thistle seed oil. More unsaturated fatty acids were identified in hemp seed oil (89–92%). Especially the content of linoleic acid was much higher in hemp seed oil compared with milk thistle seed oil. On the other hand, milk thistle seed oil was abundant in oleic acid (35–42%).

**Table 7:** The content of fatty acids in milk thistle and hemp seed oils.

Fatty acids	% of oil	
	Milk thistle seed oil	Hemp seed oil
**Free fatty acids**	Not studied	**2% w/w**
Cholesterol	Not studied	–
Tetrahydrocannabinol (THC)	Not studied	< 5 ppm
**Saturated fatty acids**	**17–20%**	**9–10%**
Lauryl (C12:0)	0.25	–
Myristic (C14:0)	1.10	–
Palmitic (C16:0)	11–13%	6.00%
Stearic (C18:0)	2–4%	2–3%
Arachidic (C20:0)	2–4%	0.8%
**Unsaturated fatty acids**	**78–85%**	**89–92%**
Oleic (C18:1n9)	35–42%	10%
Linoleic (C18:2n6)	32–36%	57%
Alpha-linolenic (C18:3n6)	3–6%	20%
Gamma-linolenic (C18:3n6)	Not studied	2–4%
Eicosanoic (C20:1)	Not studied	0.4%

### Effect of chlorogenic acid on the lipid peroxidation process in oils

**Figure 6:**
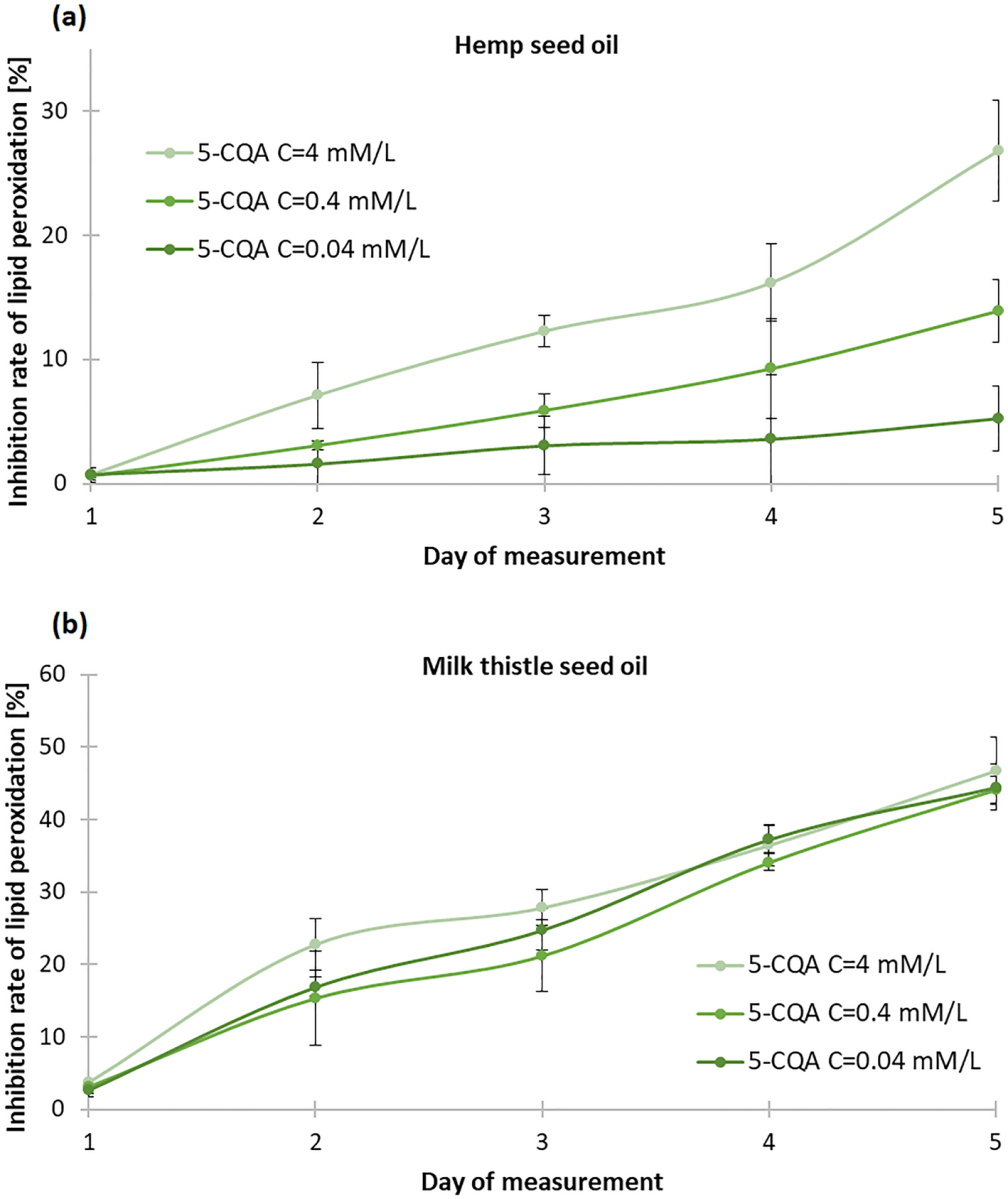
Effects of chlorogenic acid (5-CQA) on the lipid peroxidation process in (**a**) hemp seed oil, (**b**) milk thistle seed oil, depending on the concentration of 5-CQA (± standard deviation are depicted).

Because of the high content of fatty acids in the plant oils, they undergo oxidation during storage in the oxygen atmosphere. Therefore we studied the effect of addition of selected natural phenolic compound: 5-caffeoylquinic acid (5-CQA; chlorogenic acid) on the lipid peroxidation. 5-CQA was added to hemp and milk thistle seed oils in various concentrations. The 5-CQA was chosen because of its high antioxidant potential (on the basis of the above described studies), good solubility in polar and non-polar solvent and natural origin^60,61^. Literature data show that 5-CQA exhibits activity towards hydroxyl radicals, which cause lipid oxidation, thus interrupting free radical reactions during lipid peroxidation^62^. In addition, chlorogenic acid brings many pro-health benefits, including antioxidant, antibacterial, anti-inflammatory, neuroprotective, anti-cancer, antiviral and as a stimulator of the central nervous system (CNS). It is speculated that chlorogenic acid may play a key role in the regulation of lipid metabolism and thus help in the treatment of cardiovascular disease and obesity^63^. Therefore, addition of 5-CQA to the oil would not only slow down its oxidation (it would act as an antioxidant), and spoilage, but also would be a valuable pro-health food additive, making the food functional. The obtained results pointed that 5-CQA inhibited lipid oxidation and therefore the oil spoilage (Figure 6). During the five days of measurement, the inhibition of lipid peroxidation increased steadily. Increase in the concentration of 5-CQA enhanced the process – especially in the case of hemp seed oil. In the last day of measurements the percent of lipid peroxidation inhibition was: 26.822 ± 4.082%, 13.897 ± 2.547%, 5.248 ± 2.624% in the case of hemp seed oil, and 44.46 ± 0.024%, 44.12 ± 0.020%, 46.74 ± 0.039% in the case of milk thistle seed oil for the subsequent concentrations of 5-CQA.

## Conclusions

**Figure 7:**
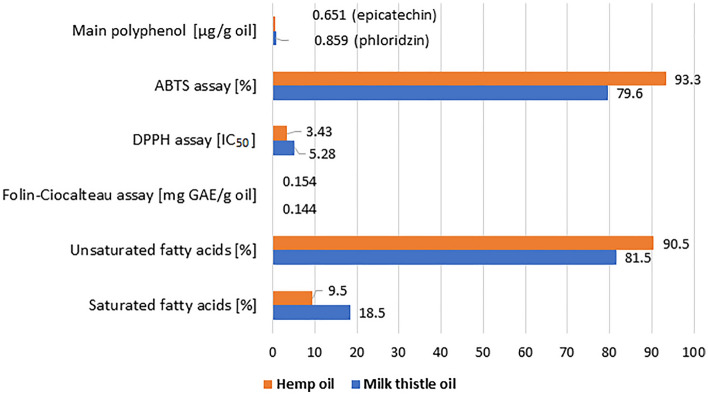
Comparison of selected parameters obtained for hemp and milk thistle seed oils (content of the main polyphenol and the results of the ABTS, DPPH and Folin–Ciocalteau assays were given for methanolic extracts).

Food products with high antioxidant potential are wanted by consumers due to their pro-health properties. In this work the hemp and milk thistle seed oils were compared for the content of phenolic compounds with antioxidant properties, fatty acids and their antioxidant activity. Testing the antioxidant properties of plant products or simple chemicals requires a sample preparation step by dissolving it in in organic solvent or water. The choice of solvent for extraction and the extraction method determine the final results. In this work methanol, ethanol and their 80% aqueous solutions were selected for simple extraction and methanol for SPE extraction—the methods and solvents most often used for the extraction of edible oils. In the case of hemp seed oil the methanolic and 80% ethanolic extracts were the most abundant in phenolic compounds. Whereas, in the case of milk thistle seed oil, the methanolic and 80% methanolic extracts were rich in phenolic antioxidants. Simple extraction was more efficient in the extraction of phenolic compounds compared to the SPE. The oils from hemp and milk thistle seeds haddifferent phenolic profiles. Hemp seed oil was reach in (-)epicatechin and catechin, and contained vanillic and ferulic acids which were not detected in the extract obtained from milk thistle seed oil. Milk thistle seed oil was abundant in phloridzin, catechin and procyanidin C1, and additionally contained quercetin, protocatechuic and syringic acids—not determined in hemp seed oil. Phloridzin, epicatechin and quercetin possessed high antioxidant properties compared to hydroxy- and methoxycinnamic acids found in the studied oils. The higher content of phenolic compounds in milk thistle seed oil compared to hemp seed oil determined by the use of HPLC method not correlated with the antioxidant potential determined by the use of ABTS and DPPH assay. Hemp seed oil showed higher antioxidant potential determined by the use of these two spectrophotometric methods probably caused by the participation of unsaturated acids in quenching the DPPH^•^ radicals and ABTS^•+^ cation radicals. The hemp seed oil was more abundant in unsaturated acids compared to milk thistle seed oil (Figure 7).

Hemp and milk thistle seed oils were rich in phenolic antioxidants and unsaturated fatty acids which are sensitive to oxidation. The addition of chlorogenic acid to these oils inhibited the oil peroxidation process, extending its service life.

## Data Availability

The data presented in this study are available on request from the corresponding author.
